# Are There Indeed Spliced Peptides in the Immunopeptidome?

**DOI:** 10.1016/j.mcpro.2021.100099

**Published:** 2021-05-20

**Authors:** Arie Admon

**Affiliations:** Faculty of Biology, Technion-Israel Institute of Technology, Haifa, Israel

**Keywords:** HLA, MHC, peptidome, immunopeptidome, peptide splicing, proteasome, LC-MS/MS, FDR, false discovery rate, HLA, human leukocyte antigen

## Abstract

The claims that a large fraction of the immunopeptidome is composed of spliced major histocompatibility complex (MHC) peptides have stirred significant excitement and raised controversy. Here, I suggest that there are likely no spliced peptides in the immunopeptidome, and if they exist at all, they are extremely rare. I base this claim on both biochemical and bioinformatics considerations. First, as a reactant in normal proteolytic reactions, water will compete with transpeptidation, which has been suggested as the mechanism of peptide splicing. The high mobility and abundance of water in aqueous solutions renders transpeptidation very inefficient and therefore unlikely to occur. Second, new studies have refuted the bioinformatics assignments to spliced peptides of most of the immunopeptidome MS data, suggesting that the correct assignments are likely other canonical, noncanonical, and post-translationally modified peptides. Therefore, I call for rigorous experimental methodology using heavy stable isotope peptides spiking into the immunoaffinity-purified mixtures of natural MHC peptides and analysis by the highly reliable targeted MS, to claim that MHC peptides are indeed spliced.

## Highlights

• Peptide splicing was suggested to contribute to the immunopeptidome.
• I suggest that this idea should be reconsidered based on new evidences.
• Both biochemical and bioinformatics considerations argue against peptide splicing.

## Graphical Abstract

**Figure:**
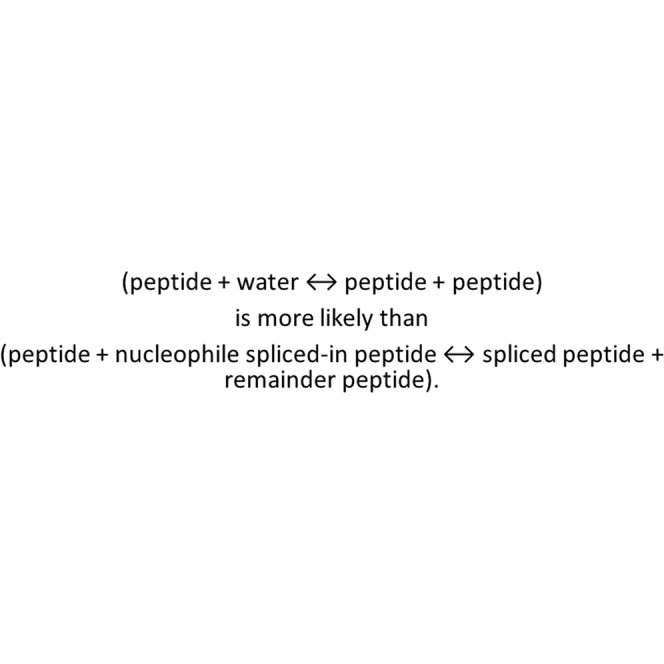

## In Brief

Peptide splicing was suggested to significantly contribute ligands to the immunopeptidome. This article argues that peptide splicing is at most very rare, even if it happens at all. Considerations against peptide splicing are based on bioinformatics calculations related to the analysis of the LC-MS/MS data, and on the abundance of water in the cells, which should compete effectively with the transpeptidation reaction, needed for peptide splicing.

The major histocompatibility complex (MHC) molecules transport peptides from the cell interior and ‘present’ them at the cell surface, enabling immune scrutiny of the health status of the cells. MHC class I molecules are expressed on most nucleated cells of vertebrates and present peptides derived from proteolysis of intracellular proteins. MHC class II molecules are expressed on immune cells and present peptides originating from intracellular proteins and from proteins taken up from outside the cells. Presentation of just a few molecules of pathogen-derived (or cancer-specific) peptides is sufficient to elicit strong immune responses, to cytotoxic killing of the diseased cells, and to stimulation, expansion, and differentiation of B cells into plasma cells, leading to the production of anti-pathogen antibodies (1, 2).

A significant degree of excitement has arisen in the field of MHC peptidome (immunopeptidome) research, with the claim that some MHC-presented peptides derive from peptide splicing (3, 4, 5), as reviewed (6, 7, 8, 9, 10, 11, 12). This concept has stirred the field tremendously; if indeed, some peptides are spliced before loading onto the MHC, the size of the required protein sequence database used for searching peptide ligands, is many folds larger than the currently used canonical protein sequence database. It would also suggest that many of the currently identified sequences of nonspliced human leukocyte antigen (HLA) peptides are incorrect. This possibility may also complicate the central dogma of immune tolerance, namely, that presentation of ‘self’ peptides in the thymus leads to deletion of the ‘anti-self’ T cells during the development of the adaptive immune system (1, 2). To enable such immune tolerance, identical peptide splicing reactions must occur both in the thymus and in the periphery (8). The proteasome was suggested as the likely site of these proteasome-catalyzed peptide splicing because it is considered the main producer of peptides for MHC presentation (13, 14, 15, 16, 17, 18) and treatment of the cells with proteasome inhibitors modulated the presentation level of the supposedly spliced peptides (3, 19, 20). The notion that proteasome-catalyzed peptide splicing produces larger pools of ligands for MHC peptidome presentation was considerably promoted after the implementation of dedicated software tools that use LC-MS/MS data to identify spliced peptides (SpliceMet) (21).

The proteasomes have been suggested to perform the splicing reaction by transpeptidation (3, 5, 22, 23, 24), with *cis*-splicing preferred over *trans*-splicing (splicing fragments of the peptides from one versus two different proteins) (23, 25). Splicing of peptides in the reverse order was also suggested (5, 26), as well as splicing with isopeptide bonds (27). Differential effects of the standard proteasomes and immunoproteasomes on peptide splicing were also demonstrated (19, 22, 28). In addition, hybrid insulin peptides were suggested to be processed in the β cell crinophagic bodies (crinosomes), where insulin degradation products are fused and then presented by MHC class II molecules, leading to diabetes and autoimmunity (11, 28, 29, 30, 31, 32). More recently, it was also suggested that fusion between human and viral peptides with subsequent splicing may increase the range of the immunopeptidomes even further, and induce immunogenic reactions that can lead to diabetes (33, 34). Furthermore, in very careful and meticulous analyses, the authors of the first publications on spliced MHC peptides (3, 4) demonstrated that only the spliced peptide sequences elicited immune responses by the T cells clones, which were oblivious to the nonspliced peptides. The immunopeptidome research community has generally related to these early findings as an exciting possibility and awaited further studies and the development of methodologies to discover the few spliced peptides among the large immunopeptidomes.

A major twist in the story of spliced HLA peptides occurred when Liepe and Mishto in the Kloetzel and Heck labs (20) performed a large-scale search for spliced MHC peptides in their own immunopeptidome LC-MS/MS data, as well as the data of Bassani-Sternberg *et al.* (35), and have shown that approximately 25% of the HLA peptidome is composed of spliced peptides. Supportive evidence was obtained using additional cultured human cell lines, primary cancer cells (36), new software tools (37, 38, 39), and cells infected with pathogens (40, 41, 42), as reviewed (8, 9, 10, 43, 44). Yet, other laboratories disagreed with the claim that a large percentage of the immunopeptidome is spliced and that many of the identifications in the canonical immunopeptidome data are incorrect. The reanalysis of the data of Bassani-Sternberg *et al.* (35) used by Liepe *et al.* (20) concluded that, at most, the percentage of spliced peptides does not exceed 2 to 6% of the canonical immunopeptidome (45). First, the spliced peptides’ sequences do not fit the canonical sequence motifs of the cells (lengths and anchor residues), as do the canonical peptide sequences identified with the same MS/MS spectra. The spliced peptide sequences had worse MS/MS identification scores than the canonical, had higher mass errors, and fitted less the synthetic peptides’ MS/MS relative to the canonical (UniProt) peptides. Furthermore, many of the MS/MS of the supposedly spliced peptides fitted better post-translationally modified canonical peptides (45). Subsequently, the published spliced peptide data of Faridi *et al.* (37) that claimed 13 to 45% of the immunopeptidome is composed of spliced peptides were reanalyzed by Rolfs *et al.* (38) using newly developed software tool (Neo-Fusion) and determined that the percentage of spliced peptides was at most 1 to 6%. The canonical peptides have better scores than the spliced peptides, and between calculated and observed chromatography retention times. Reanalysis by Lichti (46) of the data of Wan *et al.*, Bassani-Sternberg *et al.*, and Faridi *et al.* (31, 35, 37) reached similar conclusions, namely that only a small subset of the immunopeptidome is possibly spliced and that many of the previous spliced peptide identifications were actually of canonical post-translationally modified peptides (except the HLA-A∗01:01 cell line of Faridi *et al*. (37) where up to 17% of the peptides could be explained by splicing). A series of comments and responses that ensued these publications (47, 48) did not result in an agreement, and the debate is still going on (44) and becoming even more emotional. Recently, Willimsky *et al.* (49) also concluded that the frequency of spliced epitopes is largely overestimated.

As with proteomics, MHC peptides are identified by LC-MS/MS using their observed precise masses and the masses of their gas-phase generated fragments (50), followed by a comparison of the data to *in silico*–generated simulated spectra of ‘no-enzyme’ proteolysis of the known protein sequences of the animal (51, 52, 53, 54, 55, 56). When peptide splicing is considered, a significantly larger protein sequence database than the canonical should be used to include all potential spliced sequences. The larger sequence databases increase the chance of assignment of erroneous peptide sequences, even under a stringent false discovery rate (FDR) of 1%, and reduces the number of correctly identified peptides (57, 58, 59). As described above, the efforts to overcome these limitations included the development of new software tools to identify spliced peptides, such as that designed by Rolfs *et al.* (38), who evaluated different immunopeptidome LC-MS/MS datasets and came to the conclusion that indeed the percentage of the spliced peptides among the immunopeptidome is only about 1% of the identified peptidome, and by Mylonas *et al.* (45) who concluded about 2 to 6% spliced peptides. In contrast, Faridi *et al.* (37) performed a similar analysis, using their LC-MS/MS search procedure, combined with *de novo* sequencing with the PEAKS Studio software tool (60), but using a significantly smaller decoy database, and came to a similar conclusion as did Liepe *et al.* (20), namely that a large fraction of the immunopeptidome is indeed composed of spliced peptides. Importantly, Mishto *et al.* (22) analyzed peptide splicing using isolated proteasomes (of yeasts) in H_2_^18^O buffer and pools of synthetic peptides and concluded that 1 to 2% of the processed peptides are spliced. In addition, Berkers *et al.* (61), Paes *et al.* (62), and Specht *et al.* (25) used a set of synthetic peptides to elucidate the patterns and degree of peptide splicing by isolated human proteasomes, while Kuckelkorn *et al.* (63) have performed this analysis also with thymoproteasomes and immunoproteasomes, and both studies have detected a significant fraction of the peptides as being spliced peptides. Recently, Erhard *et al.* (64) performed a similar search using a new software tool (Peptide-PRISM) that also uses the PEAKS *de novo* search engine and suggested that most of the supposedly spliced peptides in the study by Liepe *et al.* (20) are actually products of canonical peptides and noncanonical translations from previously unidentified ORFs. They concluded that the percentage of spliced MHC peptides is less than 1% of the immunopeptidome. The authors provide a detailed explanation on the problem of erroneous identification of peptide sequences because of problems with assigning the correct FDR by the target-decoy approach in the Supplementary notes of Erhard *et al.* (64).

Here, I argue that peptide splicing is, at most, an extremely rare event and likely does not happen at all. I base this claim on two main arguments. The first is a biochemical consideration. For peptide splicing to be catalyzed by a protease, such as a proteasome, by transpeptidation during normal proteolysis, water must be excluded from its active site. Peptide splicing requires that the highly unstable acyl-enzyme reaction intermediate be attacked by the nucleophile N terminus of another peptide that eventually forms the spliced-in partner of the final product. During regular proteolysis, water molecules provide this nucleophile, attack the acyl-enzyme intermediate, and release the proteolysis product from the active site (8, 22, 24). The water molecules are eventually split to hydrolyze the peptide bond and contribute hydroxyl and hydrogen to each of the products. Water is (obviously) very abundant in aqueous solutions, with a concentration of about 55 M, whereas the peptide substrate candidates for splicing are present at concentrations many orders of magnitude lower, even within the proteasome cavity. It is true that within the confinement of the active site of the proteasome, the local concentration of free peptides can be much higher than their concentrations in the surrounding cytoplasm. However, the proteasome cavity is a large and open cavity, with an abundance of free water molecules (65, 66, 67). Similarly, in the beta cell granules or crinophagic bodies, the concentration of proteins and peptides can be very high, thus favoring peptide splicing (11). Therefore, to achieve effective peptide splicing, the nucleophile N terminus of another free peptide must diffuse in, and attack the bound unstable acyl-enzyme intermediate faster than the abundant and highly mobile water molecules present within the same cavity. Alternatively, the nucleophile peptides may occupy simultaneously the protease active site, with the protein substrate, thus preventing water from diffusing in. No evidence for concurrent occupation of the proteasome active sites by two peptides has been demonstrated so far. The diffusion rates of peptides and proteins are orders of magnitude lower than those of water molecules. Therefore, enzymatic reactions that have a protein (or peptide) and water as substrates, and two peptides as products (peptide + water ↔ peptide + peptide), are much more likely to occur than a splicing reaction using two peptides as substrates and producing two other peptides as products (long peptide + nucleophile spliced-in peptide ↔ spliced peptide + remainder peptide).

The second argument is based on bioinformatics considerations. Although MS is a truly advanced methodology, analysis of LC-MS/MS data is still prone to some misidentifications. The level of error in identification of spliced peptides using LC-MS/MS data is large because of the sheer size of the sequence databases used for the search, which are many folds larger than the normal protein sequence database used for the identification of normal peptides. These databases are often so large that they cannot be deposited in the public repositories and are not available for evaluation by peers (20, 36, 37, 39). Using very large sequence databases or bioinformatics tools that create large numbers of alternative sequence variants results in the identification of large numbers of erroneous identifications, even when the FDR is set to the low 1%. This problem requires employing of stringent bioinformatics and experimental routines for correct assignment of peptide sequences (45, 47, 55, 68) and thus, such ‘Extraordinary claims require extraordinary evidence’ (attributed to Carl Sagan), as reviewed in (69). The claim of a large percentage of spliced peptides within the immunopeptidome requires extraordinary validations, such as analysis of synthetic versions of the selected natural peptides (see, for example, (70)), as was indeed done in the original claims of HLA peptide splicing (3, 4, 5, 7) and in the more recent studies (20, 37, 40, 42, 45, 71) on noncanonical cryptic peptides (64). There is also a need to overcome the risk of circular arguments; if a peptide sequence is predicted from a particular MS/MS spectrum and a synthetic peptide is produced based on the predicted sequence, the synthetic peptide will likely fragment similarly to the observed natural peptide. This does not provide full proof that their sequences are identical because similar fragmentation patterns may result from similar but nonidentical sequences, such as peptides with leucine or isoleucine that have the exact same mass. Sound proof that the synthetic peptide is identical to the natural one requires additional similarities other than their MS/MS patterns. For example, both synthetic and natural peptides and their fragments should have identical chromatography retention times. Their fragmentation patterns should be identical, both with regard to the fragment composition and in the relative intensities of the fragments throughout the elution profile of their chromatography peaks. Currently, the safer way to prove such correct assignment of peptide sequences is by spiking natural peptide mixtures with heavy stable isotope-labeled synthetic peptides and demonstrating that these heavy peptides coelute and cofragment exactly like their natural peptide counterparts. Performing this analysis with unlabeled, light isotope–labeled synthetic peptides is not possible because they are indistinguishable in their masses and therefore mask the signals of natural peptides, when spiked in. To perform this analysis properly, targeted MS should be used, in which the mass spectrometers are instructed to fragment multiple times, both the natural and spiked-in heavy isotope–labeled synthetic peptides during their elution peak. These methods are commonly used for targeted proteomics (72, 73) and provide the best evidence that both natural and synthetic peptides have identical retention times and fragmentation patterns (51, 74, 75, 76, 77). However, even this approach is not completely ‘problem-free’, heavy isotope–labeled peptides are costly and not readily available and therefore not relevant for validation of thousands of potentially spliced peptides. A sequence that matches perfectly a spliced peptide might still be due to RNA splicing events, a phenomenon that can be discovered by performing Ribo-seq with ribosomes collected from the same cell culture (64). Importantly, heavy peptides should be obtained only from reputable sources, to ensure absence of even trace amounts of contaminating ‘light’ synthetic peptides that might be mistaken for the natural (spliced) peptides. Therefore, realistically, validations should only be performed with randomly selected subsets of spliced peptide candidates, using well-annotated canonical peptides as the controls.

## Conclusions

I suggest that the elegant idea of a large or even a small fraction of the MHC peptidome is spliced, is likely incorrect. While I accept that it is not possible to fully rule out the existence of some spliced peptides, the difficulty to rule out the phenomenon does not mean that they do exist. It is more likely that peptides previously thought to be spliced are actually nonspliced (canonical and noncanonical) peptides that have not yet been classified as such because of more exotic post-translational modifications (46) or noncanonical transcripts that remain to be identified (64, 78, 79). Future studies on spliced MHC peptides must resort to extraordinary validations, deposit the amino acid sequence databases used for the search, or at least provide their size, and such studies should not disregard the high abundance of water in the aqueous cytoplasm.

## Conflict of interest

The author declares no competing interests.
